# Mechanistic Insights into the Inhibition of a Common CTLA-4 Gene Mutation in the Cytoplasmic Domain

**DOI:** 10.3390/molecules29061330

**Published:** 2024-03-16

**Authors:** Jikang Xu, Yu Zhang, Lijuan Shen, Lingyu Du, Hongjuan Xue, Bin Wu, Bo OuYang

**Affiliations:** 1 State Key Laboratory of Molecular Biology, Shanghai Institute of Biochemistry and Cell Biology, Center for Excellence in Molecular Cell Science, Chinese Academy of Sciences, Shanghai 200031, China; xujikang2020@sibcb.ac.cn (J.X.);; 2 University of Chinese Academy of Sciences, Beijing 100049, China; 3 National Facility for Protein Science in Shanghai, ZhangJiang Laboratory, Shanghai Advanced Research Institute, Chinese Academy of Sciences, Shanghai 201203, Chinabin.wu@sibcb.ac.cn (B.W.)

**Keywords:** CTLA-4, G199R, NMR spectroscopy, lipid regulation, inhibitory mechanism

## Abstract

Cytotoxic T-lymphocyte antigen 4 (CTLA-4) is a pivotal immune checkpoint receptor, playing a crucial role in modulating T-cell activation. In this study, we delved into the underlying mechanism by which a common mutation, G199R, in the cytoplasmic domain of CTLA-4 impacts its inhibitory function. Utilizing nuclear magnetic resonance (NMR) spectroscopy and biochemical techniques, we mapped the conformational changes induced by this mutation and investigated its role in CTLA-4 activity. Our findings reveal that this mutation leads to a distinct conformational alteration, enhancing protein–membrane interactions. Moreover, functional assays demonstrated an improved capacity of the G199R mutant to downregulate T-cell activation, underscoring its potential role in immune-related disorders. These results not only enhance our understanding of CTLA-4 regulatory mechanisms but also provide insights for targeted therapeutic strategies addressing immune dysregulation linked to CTLA-4 mutations.

## 1. Introduction

The immune system has a pivotal role and delicate balance in maintaining an effective immune defense and preventing autoimmune reactions. Central to this balance is the tightly regulated process of T-cell activation, which involves a series of molecular checkpoints to fine-tune immune responses. Within this intricate network of immune regulation, cytotoxic T-lymphocyte antigen 4 (CTLA-4), also recognized as CD152, emerges as a pivotal checkpoint. CTLA-4 was first identified as a homologue of the co-stimulatory molecule CD28 [1], which interacts with the B7 family of ligands (B7-1/CD80 and B7-2/CD86) on antigen-presenting cells (APCs) [2,3]. Although CTLA-4 shares a high degree of structural similarity with CD28, its functional role in the immune system is distinct [4,5,6]. CTLA-4 is predominantly expressed on activated T cells, where it acts as a negative regulator of T-cell activation and effector functions [7]. Unlike CD28, most CTLA-4 molecules remain intracellular in vesicles, and only a small portion is expressed on the T-cell surface in a dynamic way [8]. Moreover, CTLA-4 has higher binding affinity to B7 molecules compared to CD28, thus engaging in a competitive interplay with CD28 [9]. 

CTLA-4 is a single-pass type I transmembrane glycoprotein belonging to the immunoglobulin superfamily. It is composed of 223 amino acids, including a 35 amino acid signal peptide. Its structure comprises three distinct domains: an extracellular immunoglobulin variable-like domain (IgV-like domain), a transmembrane domain, and a cytoplasmic domain. The cytoplasmic domain contains 36 amino acids, which is particularly intriguing due to its rich assembly of signaling motifs but lack of the classical immune receptor tyrosine based inhibitory motif (ITIM) [10,11]. Central to this domain is the tyrosine-based motif Y^201^VKM, homologous to the CD28 YMNM sequence [12], recognized for its instrumental role in transmitting inhibitory signals. Numerous intracellular proteins have been reported to engage in binding interactions with the Y^201^VKM motif [13,14,15]. Through these interactions, CTLA-4 actively mediates the attenuation of T-cell activity by recruiting and forming complexes with key signaling molecules. Furthermore, the cytoplasmic tail of CTLA-4 harbors two additional motifs of interest: the lysine-rich motif (K^188^MLKKR) and the tyrosine residue within the C-terminal Y^218^FIP motif. These motifs have also been reported to interact with kinases, thereby contributing to the regulatory functions of CTLA-4 [16,17]. The catalytic actions of these kinases, particularly the phosphorylation of specific tyrosine residues, serve as fine-tuning mechanisms for CTLA-4 signaling, offering a dynamic modulation avenue for its inhibitory potential. 

Intriguingly, while these intricate intracellular signaling mechanisms have been extensively explored, evidence from other studies suggests that only a membrane-anchored extracellular domain or a tailless version of CTLA-4, especially a C-terminal truncated mutant (ΔC), can independently induce immune suppression [18,19,20,21,22]. In contrast to their roles in signal transduction, the motifs in the cytoplasmic domain were reported to participate in endocytosis, regulating CTLA-4 levels at the plasma membrane, thereby allowing normal CD28 engagement. Specifically, the Y^201^VKM motif within this domain is known to expedite endocytosis through its interaction with the μ2 subunit of the AP-2 complex, essential for CTLA-4 internalization and recycling [23,24]. Mutations at Y201 (Y201F/G/V) have been observed to hinder CTLA-4 endocytosis, leading to increased membrane expression [9,25,26]. Moreover, the Y^218^FIP sequence is identified as an alternative adaptor in CTLA-4 endocytosis [24,27]. Thus, these studies suggest that the suppressive function of CTLA-4 is likely mediated by the extracellular domain, while the cytoplasmic domain contributes to suppressive function by controlling the protein quantity and cellular localization of CTLA-4 [28]. Overall, the intriguing discoveries of CTLA-4 immunoregulatory mechanisms have underscored the ongoing need for further investigation and a deeper understanding of its complex role in immune modulation.

In this evolving landscape, genetic mutations in the cytoplasmic domain of CTLA-4 have garnered our interest for their potential impact on immune responses. Numerous human non-synonymous single nucleotide polymorphisms (nsSNPs) have been identified in genes like CTLA-4 due to recent advancements in next-generation sequencing technologies [29,30]. These nsSNPs are of particular interest since over 50% of mutations implicated in human inherited diseases are non-synonymous variants [31]. However, the increased detection of genetic variations brings about new challenges in the interpretation of sequencing data. It underscores the critical need for the comprehensive characterization of nsSNPs, particularly their effects on protein function. The nsSNPs in CTLA-4 have been reported to potentially alter its function, which could consequently lead to dysregulated immune responses [32]. Therefore, exploring the exact role and regulatory mechanisms of CTLA-4 cytoplasmic domain mutations not only contributes to our fundamental understanding of immune regulation, but also holds significant potential for developing targeted therapies in immunology and oncology. 

To this end, our research began with an analysis of genetic mutations in the cytoplasmic domain of CTLA-4, identifying, in particular, mutations linked to cancer development. We then employed nuclear magnetic resonance (NMR) spectroscopy and biochemical assays to characterize the cytoplasmic domain of CTLA-4. This approach allowed us to gain insights into the structural and functional aspects of the CTLA-4 protein. Subsequently, we specifically examined the G199R mutation, a variant that is frequently observed but has an unclear impact on CTLA-4 inhibitory function, to compare its properties with those of the wild type. Our objective was to elucidate the regulatory mechanism of this mutation, capturing the dynamic conformational changes it induces and assessing its influence on the properties of CTLA-4. Furthermore, we validated the unique implications of the G199R mutant in regulating CTLA-4 function. Through this comprehensive analysis, we endeavored to augment our foundational knowledge of the intricacies governing CTLA-4 modulatory mechanisms, potentially contributing to the development of targeted immune therapies.

## 2. Results

### 2.1. Bioinformatic Analysis of Genetic Mutations in the Cytoplasmic Domain of CTLA-4

**Figure 1:**
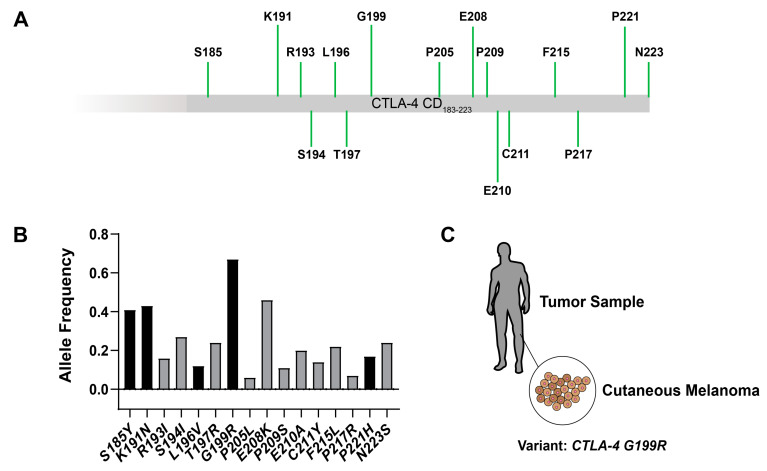
Statistics of point mutations in the cytoplasmic domain of CTLA-4 in tumor samples from cBioPortal data sets for cancer genomics. (**A**) Point-mutated amino acids in the cytoplasmic domain of CTLA-4 according to data sets. The length of the green lines shows the quantity level of the mutations (tumor samples). (**B**) Allele frequency of the point mutations in the cancer samples. Black and grey columns show the study origin from TCGA PanCancer atlas and other sources (MSK, etc.), respectively. (**C**) Model of point mutation G199R from cutaneous melanoma tumor samples.

First, we directed our focus towards the investigation of genetic mutations in the cytoplasmic domain of CTLA-4 associated with cancer. Using cancer genomic datasets from cBioPortal [33,34,35], we identified a series of point mutations in the cytoplasmic domain of CTLA-4, detected in tumor samples. These mutations spanned various residues, notably including S185Y, K191N, R193I, S194I, T197R, G199R, P205L, E208K, among others (Figure 1A). To further comprehend the prevalence and distribution of these mutations across different cancers, we conducted an allelic frequency analysis, drawing data from diverse sources, such as The Cancer Genome Atlas (TCGA) PanCancer Atlas and Memorial Sloan Kettering Cancer Center (MSK) [36,37]. The results of our analysis demonstrated that the G199R mutation found in TCGA-DA-A1HV and SP82900 samples [36,38], which might be highly relative to cutaneous melanoma, exhibited the highest frequency within the CTLA-4 cytoplasmic domain (Figure 1B,C). However, the functional consequences of this mutation on CTLA-4 activity and its potential role in tumor survival remain to be fully understood. 

### 2.2. Membrane Interaction of the Cytoplasmic Domain of CTLA-4 in the Presence of Acidic Lipids

We initiated our investigation by characterizing the cytoplasmic domain of human CTLA-4 residues A183-N223 (referred to as CD_183–223_). We first expressed the codon-optimized CD_183–223_ within an *E. coli* expression system. CD_183–223_ was strategically fused to the C-terminus of an 8×His-MBP tag, with a PreScission protease (3C) cleavage site inserted between them (Figure S1A). Subsequently, the fusion protein underwent a purification process involving nickel affinity chromatography, followed by sequential cleavage by 3C protease and reverse-phase high performance liquid chromatography (HPLC) to remove the 8× His-MBP tag (Figure S1B). The identification and purity of CD_183–223_ were confirmed by sodium dodecyl-sulfate polyacrylamide gel electrophoresis (SDS-PAGE) (Figure S1C) and mass spectrometry (Figure S1D).

In order to investigate CD_183–223_-membrane interactions, the purified protein was subjected to incubation with liposomes composed of distinct lipid compositions referred to in a previous study [39], including 100% 1,2-dimyristoyl-*sn*-glycero-3-phosphocholine (DMPC), 25% 1,2-dimyristoyl-*sn*-glycero-3-phosphoglycerol (DMPG), or 25% 1′,3′-*bis* [1,2-dimyristoyl-*sn*-glycero-3-phospho]-glycerol (cardiolipin) supplemented with 75% DMPC, 100% 1-palmitoyl-2-oleoyl-glycero-3-phosphocholine (POPC), and 25% 1-palmitoyl-2-oleoyl-*sn*-glycero-3-phospho-(1′-*rac*-glycerol) (POPG) supplemented with 75% POPC, 100% L-α-phosphatidylethanolamine (PE), and 50% L-α-phosphatidylinositol (PI) supplemented with 50% PE. Following the ultracentrifugation, CD_183–223_ was observed to be associated with liposomes containing the acidic phospholipids DMPG, POPG, cardiolipin, and PI, while no association was detected with the zwitterionic phospholipids DMPC, POPC, and PE (Figure S1E). These findings substantiate the requirement of acidic phospholipids for the membrane binding of CD_183–223_.

**Figure 2:**
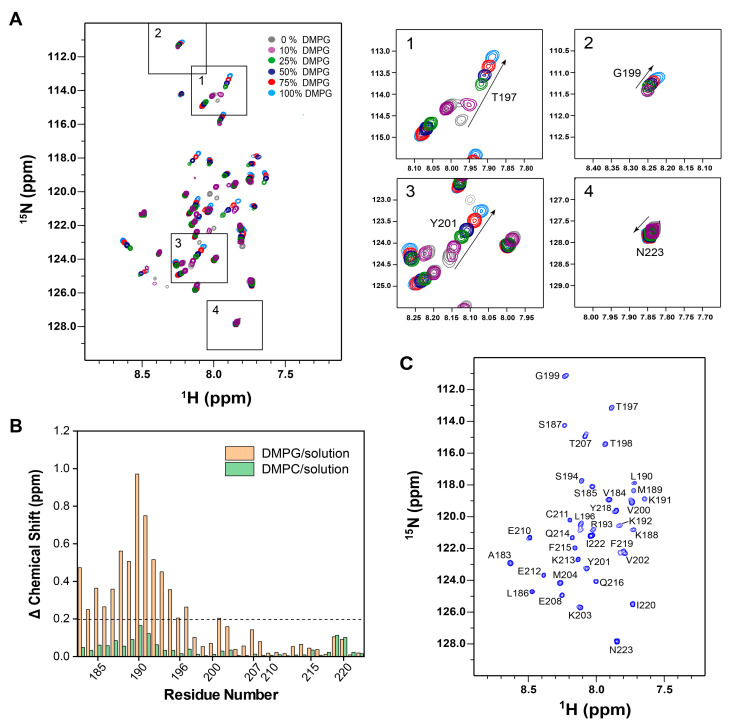
NMR characterization and lipid effects on CD_183–223_. (**A**) Superimposed 2D ^1^H-^15^N TROSY-HSQC spectra of CD_183–223_ in 100% DMPC/DH^6^PC (grey), 10% DMPG-90% DMPC/DH^6^PC (purple), 25% DMPG-75% DMPC/DH^6^PC (green), 50% DMPG-50% DMPC/DH^6^PC (cyan), 75% DMPG-25% DMPC/DH^6^PC (red), and 100% DMPG/DH^6^PC (blue) bicelles. The q values for the bicelles are all 0.7. The right panels 1–4 show the same spectral regions labeled on the full spectrum, highlighting the chemical shift changes for T197, G199, Y201, and N223, respectively. (**B**) Comparison of site-specific amide backbone ^15^N NMR chemical shift changes of CD_183–223_ between DMPC/DH^6^PC bicelles versus the solution (green) and DMPG/DH^6^PC bicelles versus the solution (orange). (**C**) ^1^H-^15^N TROSY-HSQC spectrum of CD_183–223_ in DMPG/DH^6^PC bicelles with backbone resonances assigned. The spectrum was recorded at ^1^H frequency of 600 MHz using [^15^N, ^13^C]-labeled protein.

Subsequently, we applied solution NMR spectroscopy to further delineate the interaction between CD_183–223_ and the membrane, following a previously reported protocol [40]. The backbone resonance assignments of CD_183–223_ in buffer solution were achieved through a standard suite of triple resonance experiments (Figure S2A). ^15^N-labeled CD_183–223_ was then reconstituted into bicelles composed of zwitterionic DMPC and 1,2-dihexanoyl-*sn*-glycero-3-phosphocholine (DH^6^PC) at a molar ratio of q = 0.7. Subsequently, we altered the lipid composition by incorporating varying proportions of DMPG into the DMPC/DH^6^PC bicelles (Figure 2A). Two-dimensional (2D) ^1^H-^15^N transverse relaxation optimized spectroscopy (TROSY) experiments were employed to monitor chemical shift perturbations. Notably, CD_183–223_ in DMPC/DH^6^PC bicelles (q = 0.7) exhibited little detectable chemical shift differences compared to the protein in buffer solution (Figure 2B and Figure S2B). Conversely, CD_183–223_ in acidic DMPG/DH^6^PC bicelles (q = 0.7) displayed significant chemical shift alterations and changes in peak intensities in the majority of N-terminal resonance peaks (Figure 2B and Figure S2C,D). These results strongly suggested the specific binding of CD_183–223_ to acidic phospholipids within the bicelles.

### 2.3. Membrane Partition of WT in DMPG Bicelles

We next studied how CD_183–223_ interacts with the membrane using solution NMR spectroscopy. As mentioned above, a series of NMR spectra at different proportions of DMPG/DMPC showed that the chemical shifts of the N-terminal resonances moved continuously (Figure 2A), indicating that CD_183–223_ exists in a two-state transition from a membrane-unbound to a membrane-bound status. Intriguingly, the residues T198-V200 exhibited few interactions with the membrane compared to its neighboring residues (Figure 2 and Figure S2C). In an effort to stabilize CD_183–223_ in its membrane-associated conformation, a lipid mixture of 100% DMPG/DH^6^PC at q = 0.7 was selected for subsequent protein–membrane interaction studies. Complete backbone resonance assignments were accomplished through a standard set of triple resonance experiments for this membrane-bound system (Figure 2C). The secondary structures of CD_183–223_ were derived from analysis of the backbone chemical shifts using the TALOS+ program (Figure S2E) [41]. Interestingly, the residues A183-L190 exhibited an alpha-helical conformation within the context of 100% DMPG/DH^6^PC bicelles. However, these same residues adopted an unstructured configuration when examined in solution. This observation suggests that the presence of negatively charged lipids induced significant conformational changes within the N-terminal region of the cytoplasmic domain.

To further understand the protein conformational behavior in relation to membrane binding, we conducted an analysis using paramagnetic relaxation enhancement (PRE) techniques. Specifically, we used the lipophilic paramagnetic probe 16-DSA (16-DOXYL stearic acid) and hydrophilic paramagnetic probe Gd-DOTA (gadolinium (III) 1,4,7,10-tetraazacyclododecane-1,4,7,10-tetraacetate) to assess the depth of immersion of the protein within the lipid bilayer region of the bicelles (Figure S3A), following established procedures [42]. The intensities of residues in the N-terminal region (e.g., V184) obviously decreased as the concentration of 16-DSA increased, suggesting their deeper insertion into the lipid bilayer. Conversely, C-terminal residues, such as Q216, showed a more gradual decrease in peak intensity, indicative of their location predominantly in the aqueous phase (Figure S3B). The residue-specific paramagnetic relaxation enhancement (PRE) amplitudes, derived from the interaction with 16-DSA, further substantiate the conclusion that the N-terminus of CD_183–223_ is embedded within the lipid bilayer, while the C-terminus remains largely solvent-accessible. This conclusion is supported by lower PRE amplitudes for the C-terminal residues (Figure S3C), a pattern that aligns with data from DMPG titrations. Specifically, residues adjacent to the Y^201^VKM motif, T198-V200, exhibited weak interactions with the acidic lipid bilayer, while neighboring residues demonstrated more robust interactions with the membrane (Figure 2B and Figure S3D). These results were consistent with complementary PRE titrations of Gd-DOTA (Figure S3E,F). In addition, the residues Y218-I220 showed a weak reassociation with the lipid bilayer (Figure 2B and Figure S3D,F), which might be due to the hydrophobic interaction of the Y^218^FIP motif with the membrane, suggesting that certain motifs within the protein sequence dictate the depth and nature of membrane association.

### 2.4. Characterization of G199R by NMR Spectroscopy

**Figure 3:**
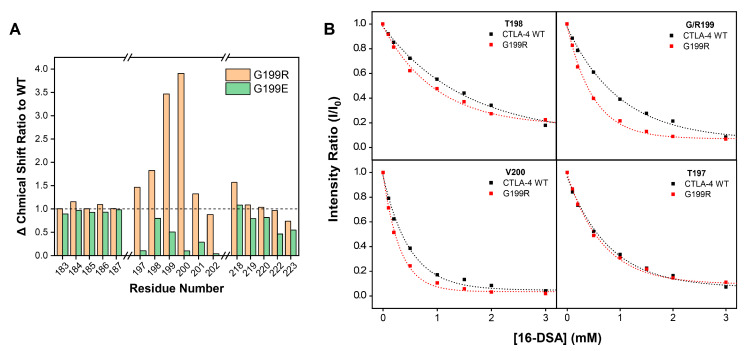
Analysis of the membrane partition of WT and G199 mutations in lipid bicelles. (**A**) The Δ chemical shift ratio of G199R (yellow) and G199E (green) to WT in DMPG/DH^6^PC bicelles versus in buffer solution. (**B**) Comparation of G199 and R199 decay curves during 16-DSA titration in the WT (black) and G199R mutation (red).

The above NMR data showed that the G199 residue, situated within the T198-V200 region and proximate to the essential Y^201^VKM motif, exhibited distinct behavior in protein–membrane interactions. Moreover, the higher occurrence of the G199R mutation in cancer samples further increased our interest in exploring its implication in an in-depth analysis. To understand the functional consequences of the G199R mutation, the G199R mutant of CTLA-4 was first constructed and subjected to biophysical analyses in comparison to the wild-type (WT) protein. Two-dimensional TROSY spectra were recorded in solution and in the presence of DMPG/DH^6^PC bicelles under the same conditions of WT. We observed significant chemical shift changes in certain amino acid residues between G199R and WT, thus backbone resonance assignments of G199R were further accomplished (Figure S4). These shifts were particularly notable in the residues K192-R193 and L196-Y201 adjacent to the site of mutation. Meanwhile, the chemical shift changes of G199R between DMPG/DH^6^PC bicelles and solution were more significant than that of WT, especially in the residues L196-Y201 and Y218 (Figure 3A), suggesting the enhanced interaction of acidic lipids in the G199R mutant. In contrast, the mutation of Gly to the negatively charged amino acid Glu (G199E) exhibited distinctive spectral characteristics when subjected to similar analyses. The G199E variant displayed minimal resonance shifts compared to its G199R counterpart, indicating the important influence of electrostatic interaction in membrane association (Figure 3A).

Subsequent to the chemical shift perturbation analysis, a 16-DSA titration experiment was conducted to probe the interaction of the G199R mutant within membrane mimetics. The results of this titration showed a faster decay rate and higher PRE_amp,_ particularly in the residues T198-Y201, than WT, indicating that the interactions between these residues and the membrane increased under the influence of the G199R mutation (Figure 3B). This is likely due to the enhanced electrostatic interactions between the positively charged arginine residue and negatively charged phospholipids. These findings collectively suggest that the G199R mutation fosters a stronger association with the membrane, potentially modulating CTLA-4 inhibitory signal transduction.

### 2.5. Functional Investigation of the G199R Mutant

**Figure 4:**
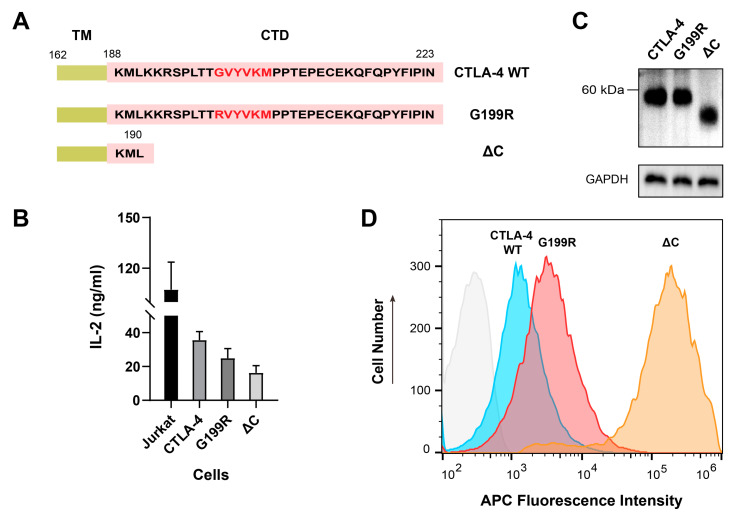
Biochemical and functional comparison between the WT, G199R, and ΔC mutant. (**A**) Amino acid sequence of the cytoplasmic domain of CTLA-4 and G199R mutant. Red letters indicate the tyrosine-containing motifs (Y^201^VKM) that are conserved between species. Domains: TM, transmembrane; CTD, cytoplasmic domain. (**B**) Bar graph summarizing IL-2 release from a 24-h Jurkat-Raji coculture with different transduced CTLA-4 mutants into Jurkat cells. The data shown are representative of the results from at least three independent experiments. (**C**) Cellular level of CTLA-4 or mutants in Jurkat cells determined by western blot. Samples were run under non-reducing conditions. (**D**) Surface level of the WT (blue), G199R (red), and ΔC mutant (yellow) in Jurkat cells determined by flow-cytometric analysis using APC-conjugated anti-CTLA-4 mAb. The control (grey) corresponds to cells without any antibody treatment.

Following our examination of the structural impact of the CTLA-4 G199R mutation, we probed its functional consequences on signaling transduction. To this end, we utilized overexpression systems in Jurkat T cells, introducing CTLA-4 WT, the G199R mutant, and a C-terminal truncated variant (ΔC) (Figure 4A). We investigated the functional impact of the G199R mutation by measuring interleukin-2 (IL-2) secretion from Jurkat cells activated by Raji B cells, using an enzyme-linked immunosorbent assay (ELISA). The results indicated that the G199R mutation enhanced the CTLA-4 inhibitory effect on IL-2 secretion compared to CTLA-4 WT, but to a lesser extent than the ΔC mutant (Figure 4B). Western blot analysis revealed no significant difference in total protein expression levels between the G199R mutant and CTLA-4 WT (Figure 4C), suggesting that the mutation does not affect CTLA-4 expression. Flow cytometry, however, demonstrated a slight increase in cell-surface expression of the G199R mutant, although not as pronounced as with ΔC (Figure 4D).

**Figure 5:**
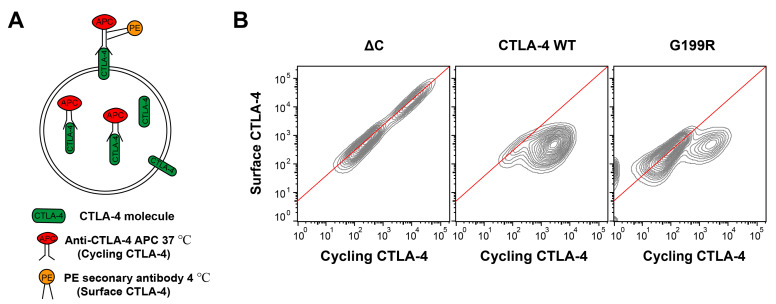
Endocytosis analysis of CTLA-4 for the WT, G199R, and ΔC mutant. (**A**) Diagram of the antibody labeling strategy for flow cytometry experiments in (**B**). (**B**) Jurkat cells expressing different CTLA-4 were labeled with anti-CTLA-4 APC at 37 °C for 30 min, followed by labeling surface CTLA-4 on ice (4 °C) with a fluorescently conjugated anti-mouse secondary antibody (PE) for 1 h. Cells were then analyzed by flow cytometry.

To understand the role of G199R in CTLA-4, we utilized flow cytometry with an APC-labeled anti-CTLA-4 antibody to track CTLA-4 trafficking through the plasma membrane over a 30-min interval at 37 °C. This allowed us to identify a pool of CTLA-4 that cycled between the plasma membrane and the cell interior. A second staining step at 4 °C with a PE-conjugated secondary antibody was employed to label and identify cell surface-resident receptors exclusively (Figure 5A). For the non-endocytic ΔC mutant [43], a linear relationship was observed between the staining at 37 °C (indicative of cycling CTLA-4) and the staining at 4 °C (indicative of surface-localized CTLA-4) (Figure 5B, red line). This observation aligns with our expectations, as receptors labeled with anti-CTLA-4 at 37 °C remain on the cell surface and are, therefore, accessible for labeling by the PE secondary antibody at 4 °C. In contrast, CTLA-4 WT displayed a clear deviation from this linear trend, indicative of receptor internalization. When staining for surface-localized CTLA-4 at 4 °C, almost all of the labeled cycling CTLA-4 is internalized at 37 °C and hence not detectable. The G199R mutant exhibited an intermediate phenotype, with most labeled proteins aligning with the expected linear relationship, but a subset veering off significantly, suggesting a reduction in endocytosis (Figure 5B). These observations collectively suggest that the G199 mutation in CTLA-4 could not only impact its cell-surface expression levels but also influence its internalization dynamics, potentially affecting its regulatory role in T-cell signaling. Further investigations are warranted to elucidate the functional implications of these findings.

## 3. Discussion

The modulation of immune responses by CTLA-4 is a complex and finely tuned process. Genetic variations within the CTLA-4 gene, and the subsequent functional implications of these mutations, have remained largely obscure in the immune response. In this study, we focused on a common mutation, G199R, situated in the cytoplasmic domain of CTLA-4. Our approach utilized a combination of NMR spectroscopy and biochemical techniques to systematically characterize the conformational and functional changes conferred by the G199R mutation. Our results indicate that the G199R mutation appears to facilitate enhanced interactions between CTLA-4 and the plasma membrane. Furthermore, functional assays complemented our structural insights by showing that the G199R mutation indeed leads to a more potent inhibition of T-cell activation. This result underscores the general importance of membrane association in functional integrity, resonating with known regulatory mechanisms of protein–membrane interactions among type I transmembrane proteins, including CD3-ε/ζ and CD28 [40,44], as well as our previous findings on PD-L1 [39].

The enhanced inhibition effect of G199R could be attributed to two mechanisms: Firstly, the increased membrane association may stabilize CTLA-4 at the cell surface, thereby reinforcing its competitive binding with CD28 for shared ligands. Empirical evidence supporting this mechanism is provided by flow cytometric analyses (Figure 4D), which demonstrate an elevated presence of the G199R variant on the membrane surface compared to the WT counterpart. Secondly, the enhanced membrane interaction of G199R may disrupt the interaction of the adjacent Y^201^VKM motif with the μ2 of AP-2 complex, potentially leading to diminished endocytosis and sustained membrane residency. The second mechanism aligns well with previous surface plasmon resonance (SPR) and structural analysis that the CTLA-4/AP-2 μ2 interaction extends beyond the Y^201^VKM motif to adjacent residues, like G199 [45]. It is also consistent with the earlier report that the truncation of the C-terminal end of CTLA-4 intensifies its inhibitory action by eliminating the Y^201^VKM motif, leading to the attenuation of endocytosis [21,22]. Additionally, considering the broad spectrum of CTLA-4 mutations in the cytoplasmic domain, such as S185Y, R193I, S194I, T197R, G199R, P205L, E208K, E210A, and P217R, which are very likely to enhance membrane interactions, it is plausible that they may share a common underlying mechanism with G199R. Conformational alterations induced by several other mutations have been investigated. Nonetheless, due to current technical limitations, distinguishing the subtle changes in membrane interactions attributable to these mutations remains a challenge, leaving the functional consequences of other CTLA-4 mutations largely unexplored. Future studies should investigate how other mutations in the CTLA-4 gene affect its interaction with the plasma membrane and downstream immune response modulation. This would involve developing more sensitive and precise techniques to detect subtle changes in membrane interactions and protein conformation induced by different mutations. Moreover, implementing quantitative assays, like the trans-endocytosis assay, could enhance our understanding of the biological relevance of these mutations and their impact on endocytosis [46].

From a therapeutic angle, with the increasing use of immune checkpoint inhibitors in cancer treatment, understanding how specific mutations can affect the function of receptors like CTLA-4 is critical. Our exploration of a distinct mechanism in the CTLA-4 cytoplasmic domain significantly advances our understanding of this crucial receptor. In particular, the enhanced inhibitory effect of the G199R mutant underscores the potential for personalized medicine approaches, where genetic profiles of patients could guide the use of immune-modulating therapies. Moreover, autoimmune conditions, where CTLA-4 function is often compromised, could benefit from insights gained from the effects of this mutation. For instance, the G199R variant may be a candidate for gene therapy approaches aimed at restoring normal immune regulation in autoimmune diseases. However, our understanding of how these mutations translate into clinical outcomes, particularly in the context of autoimmune diseases and cancers, remains limited. Future research should aim to correlate specific CTLA-4 mutations with clinical phenotypes, potentially leading to more targeted and effective therapies. This could involve extensive genotype–phenotype correlation studies and clinical trials to evaluate the efficacy of therapies tailored to specific CTLA-4 mutations.

In summary, by elucidating the subtle yet impactful changes in membrane association in CTLA-4 through NMR and biochemical analyses, our study has shed light on the intricate mechanisms by which CTLA-4 mediates immune checkpoint pathways and offers a foundation for future research into the therapeutic exploitation of CTLA-4 mutations.

## 4. Materials and Methods

### 4.1. Reagents and Cells

Lipids and detergents (DMPC, DMPG, POPC, POPG, DH^6^PC, Cardiolipin, Soy PI, and Egg Trans PE) were from Avanti Polar Lipids. Stable isotopes for NMR spectroscopy experiments were from Cambridge Isotope Laboratories. Anti-human CTLA-4 antibodies (96399) were from Cell Signaling Technology. Anti-rabbit IgG-HRP (ab97051) antibodies were obtained from Abcam. APC anti-CTLA-4 antibodies (B359454) and PE anti-mouse IgG1 (B371903) antibodies were purchased from Biolegend. GAPDH polyclonal antibodies (10494-1-AP) were obtained from Proteintech. The *E. coli* strain BL21 (DE3) and DH5α were from New England Biolabs. The HEK293FT cell line was a gift from Liming Sun (CEMCS). Jurkat T cells and Raji B cells were gifts from Chenqi Xu (CEMCS). Oligomers of constructs in this research are shown in Table S1.

### 4.2. Expression and Purification of CD_183–223_ and Its Mutants

The DNA fragment corresponding to *Homo sapiens* CTLA-4 (UniprotKB: P16410) was synthesized by GenScript (Piscataway, NJ, USA). The cytoplasmic domain of human CTLA-4 (residues 183–223), named CD_183–223_, was fused to an N-terminal 8× His tag, a maltose binding protein (MBP) protein, and a PreScission protease (3C) in the pET28a vector. The construct was transformed into BL21 (DE3) cells and grown at 37 °C in M9 media supplied with stable isotopes (^15^N, ^13^C), according to experimental requirements. When the culture reached an optical density at 600 nm (OD_600_) of 0.7–0.9, cells were cooled to 20 °C before induction with 0.2 mM isopropyl β-D-thiogalatopyranoside (IPTG) at 20 °C overnight.

The expressed fusion protein was extracted and purified by nickel affinity resins (Thermo Fisher/Cytiva, Amersham, UK) and then cleaved by 3C protease at 4 °C for 14–16 h to remove the His and MBP tag. The CD_183–223_ protein was further purified by reverse-phase HPLC with a Zorbax 300SB-C3 PrepHT column (Agilent, Santa Clara, CA, USA) using an elution gradient from 20% (*v*/*v*) acetonitrile with 0.1% (*v*/*v*) trifluoroacetic acid (TFA) to 75% (*v*/*v*) acetonitrile with 0.1% (*v*/*v*) TFA. The fractions corresponding to pure CD_183–223_ peptide were collected, lyophilized, and identified by MALDI-TOF mass spectrometry and SDS-PAGE analysis. All mutants were expressed and purified following the same procedures.

### 4.3. Liposome-Binding Assays

Mixed phospholipids were dissolved in HFIP with indicated compositions (10 mM). The solvent was evaporated under nitrogen stream to achieve a thin film. Followed by overnight lyophilization, CD_183–223_ (100 μM) was dissolved in 500 μL extrusion buffer (25 mM MES, pH 6.5, 10 mM DTT) and added to the dried lipid mixture. Liposomes were generated by ultrasonication and hydrated lipids were extruded 20–30 times using the Mini-Extruder device (Avanti Polar Lipids Inc., Alabaster, AL, USA) through a 0.2 μm polycarbonate filter (610005, Avanti Polar Lipids Inc.), until OD_600_ of the liposomes reached ≤0.4. Samples were then centrifuged at 4 °C for 1 h at 100,000× *g*. The supernatant and pellet fractions were separated and analyzed by SDS-PAGE.

### 4.4. Reconstitution of CD_183–223_ into Bicelles

The NMR samples in buffer solution were prepared by directly dissolving lyophilized proteins of CD_183–223_ and variants (1.2–1.5 mg) in 25 mM MES (pH 6.5), 5 mM DTT, 10% D_2_O. To reconstitute CD_183–223_ and variants in bicelles, 1.2–1.5 mg lyophilized proteins were mixed with ~11 mg DMPG or DMPC and dissolved in hexafluoroisopropanol (HFIP). The mixture was slowly dried to form a thin film under nitrogen stream, followed by overnight lyophilization. The dried thin film was redissolved in 0.5 mL of 25 mM MES buffer (pH 6.5) containing ~43 mM DH^6^PC. The DMPG:DH^6^PC or DMPC:DH^6^PC ratio was measured by one-dimensional (1D) NMR to verify the q value (~0.7). The final NMR sample contained 0.5–0.6 mM CTD or variants, ~30 mM DMPG or DMPC, ~43 mM DH^6^PC, 25 mM MES (pH 6.5), 5 mM DTT, and 10% D_2_O.

### 4.5. Assignment of NMR Resonances and Secondary Structure Calculation

All CD_183–223_ NMR spectra were acquired at 30 °C by VnmrJ Biopack on Agilent DD2 spectrometer (700 or 600 MHz) equipped with triple-resonance cold probes. All NMR data were processed using NMRPipe [47] and analyzed by CARA-XEASY [48] and CcpNmr [49]. Sequence-specific assignment of backbone amide resonances was performed using 0.7 mM U-[^15^N, ^13^C] CD_183–223_ or variants in protein samples in DMPG:DH^6^PC bicelles (q = 0.7) and accomplished by performing a series of standard triple resonance experiments, including the TROSY version of HNCA, HN(CO)CA, HN(CA)CO, HNCO, and HNCACB on a (^15^N, ^13^C)-labeled sample at ^1^H frequency of 600 MHz. The sequence-specific assignment of backbone resonances was determined using XEASY program. The assigned backbone chemical shift values (^15^N, ^13^Cα, ^13^C’, and ^13^Cβ) from bicelle-reconstituted samples were used as the input for TALOS+ program to predict backbone dihedral angles and to calculate secondary Cα shifts and derive the secondary structure of CD_183–223_ or variants [41].

### 4.6. PRE Titration

We took a previously developed paramagnetic probe titration method using paramagnetic agents (16-DSA and Gd-DOTA) to determine the membrane insertion of proteins in bicelles [42]. For 16-DSA and Gd-DOTA titrations, we prepared 0.7 mM ^15^N-labeled CD_183–223_ reconstituted in DMPG bicelles with q = 0.7. In brief, a stock solution of the lipophilic paramagnetic agent 16-DSA (Sigma-Aldrich, Burlington, MA, USA) (50 mM) was dissolved in bicelles with the same NMR sample buffer to prevent changes in q value in the bicelles upon addition of the titrant. The progress of the titration was monitored by measuring a set of 2D ^1^H-^15^N TROSY-HSQC spectra on 700 MHz Agilent spectrometer at each of the following 16-DSA concentrations: 0, 0.1, 0.2, 0.5, 1, 2, 3, 4, and 5 mM. As for the water-soluble paramagnetic agent Gd-DOTA, it was dissolved in NMR buffer to 500 mM and then titrated into the bicelle sample to different final concentrations: 0, 1, 2, 3.5, 6, 10, 15, 20, and 30 mM. The recovery delay was set to 3.5 s. The residue-specific PRE is defined as the ratio of peak intensity in the presence (I) and absence (I_0_) of the paramagnetic agents. Peak intensities were analyzed using CcpNmr [42,49]. The residue-specific PRE amplitude was analyzed using exponential decay fit by Origin with the following Equation (1) [42], in which τ is the decay constant and [PA] is the concentration of the paramagnetic agents:

(1) $$\frac{I}{I_{0}}{=1\;-\;\mathrm{PRE}}_{\mathrm{amp}}\left({1-\;e}^{-\;\frac{\left[\mathrm{PA}\right]}{\tau }}\right)$$

PRE_amp_ is the indicator of PRE effects of the probe on the protein, as the higher PRE_amp_ values indicate the protein is closer to the PRE probe.

### 4.7. Lentivirus Production and Transduction

For Raji B cell-mediated T-cell stimulation assays, human CTLA-4 and other variants were expressed in Jurkat cells via lentiviral transduction. To produce lentiviruses, the CTLA-4 cDNA was cloned into the pHAGE vector containing a C-terminal IRES-ZsGreen tag (a gift from Chenqi Xu), and co-transfected with the envelop plasmid pMD2.G and the packaging plasmid psPAX2 into HEK293FT cells using lipofectamine 2000 (Thermo Fisher) in DMEM medium. Virus supernatants were harvested at 48 and 72 h after transfection and filtered using a 0.45 μm filter. Before transducing, 5 × 10^5^ Jurkat cells were incubated for 6 h at 37 °C/5% CO_2_ in fresh complete RPMI medium. Virus supernatants were added to Jurkat cells and incubated for 48 h followed by normal cell culture passage. Transduced cells were sorted out via fluorescence-activated cell sorting (FACS) at least one week after lentiviral transduction.

### 4.8. Jurkat Stimulation Using Raji B Cells and IL-2 Assays

For IL-2 secretion assays, 1 × 10^5^ Raji B cells were preloaded with 30 ng/mL SEE for 30 min at 37 °C. Two × 10^5^ serum-starved Jurkat cells were co-pelleted with SEE-treated Raji B cells in a 96-well plate in triplicate wells at 37 °C/5% CO_2_, and the supernatants were centrifuged and collected after 12 h. IL-2 concentrations were quantified by ELISA using Human IL-2 ELISA MAX Deluxe (431804, Biolegend, San Diego, CA, USA).

### 4.9. Western Blot and Immunoprecipitation

For western blot analysis, equal numbers of cells were washed with PBS and then lysed in a RIPA buffer [150 mM NaCl, 1% Triton X-100, 2.5 mM sodium pyrophosphate, 1 mM β-glycerophosphate, 1 mM EDTA, and 50 mM Tris-HCl (pH 7.4)], supplied with 1 mM phenylmethanesulfonylfluoride (PMSF) and protease inhibitor cocktail (B14001, Bimake, Houston, TX, USA). Proteins were resolved by SDS-PAGE, and protein transfer was performed for 60 min at 300 mA to polyvinylidene difluoride membranes (IPVH00010, Merck Millipore, Darmstadt, Germany). After blocking with 5% skimmed milk powder in TBS-T buffer (50 mM Tris-HCl, 1.37 mM NaCl, and 2.7 mM KCl at pH 8.0 with 0.05% Tween 20) for 1 h at room temperature, the membrane was incubated with antibodies (CTLA-4 antibody (Rabbit, 96399), 1:1000; GAPDH antibody, 1:5000) overnight at 4 °C. The membrane was washed three times with TBS-T buffer and then incubated with HRP-conjugated goat anti-rabbit IgG antibodies (1:5000) for 2 h at room temperature. After washing three times with TBS-T buffer, protein bands were detected using an ECL Western blotting substrate (SB-WB012, ShareBio, Shanghai, China) and analyzed using ImageJ 1.50 software.

### 4.10. Flow Cytometry for Detection of CTLA-4

For CTLA-4 detection on cell surfaces, cells were collected and washed three times with PBS. Cells were then resuspended in 200 μL of PBS and incubated with an APC-conjugated anti-human CTLA-4 antibody (1:50) at 4 °C for 1 h. After washing three times with PBS, stained cells were analyzed using flow cytometry (BD, LSRFortessa, London, UK). Data were analyzed with FlowJo 10.5 software.

For the analysis of surface-to-cycling ratios CTLA-4, cells were first incubated with APC anti-CTLA-4 (BioLegend, San Diego, CA, USA) for 30 min at 37 °C. Cells were then placed on ice and washed 3 times with PBS (4 °C). Surface CTLA-4 was then labeled by incubation on ice with a PE anti-mouse secondary antibody for 1 h. Cells were then washed and analyzed by flow cytometry.

## Data Availability

The original contributions presented in this study are included in the article and Supplementary Materials; further inquiries can be directed to the corresponding author.

## Supplementary Materials

The following supporting information can be downloaded at https://www.mdpi.com/article/10.3390/molecules29061330/s1. Figure S1: Expression and purification of CD_183–223_; Figure S2: The NMR characterization in DMPC/DH^6^PC bicelles and solution, and the lipid composition effects on CD_183–223_; Figure S3: PRE analysis of CTLA-4 CD_183–223_ partition into lipid bicelles; Figure S4: The NMR spectra of G199R mutant in bicelles; and Table S1: List of oligomers.
