# Supplementary material for: Mechanistic Insights into the Inhibition of a Common CTLA-4 Gene Mutation in the Cytoplasmic Domain
Source: Molecules. 2024 Mar 16;29(6):1330. doi: 10.3390/molecules29061330 (PMC10974916; doi:10.3390/molecules29061330)
Supplement: Supplementary file 1 [file molecules-29-01330-s001.zip › molecules-2884591-supplementary.pdf]

Supplementary Materials

Mechanistic Insights into the Inhibition of a Common CTLA-4 Gene Mutation in the Cytoplasmic Domain

Jikang Xu [1,2], Yu Zhang [1,2], Lijuan Shen [1], Lingyu Du [1], Hongjuan Xue [3], Bin Wu [3] and Bo OuYang [1,2,*]

[1] State Key Laboratory of Molecular Biology, Shanghai Institute of Biochemistry and Cell Biology, Center for Excellence in Molecular Cell Science, Chinese Academy of Sciences, Shanghai 200031, China; xujikang2020@sibcb.ac.cn (J.X.)
[2] University of Chinese Academy of Sciences, Beijing 100049, China
[3] National Facility for Protein Science in Shanghai, ZhangJiang Laboratory, Shanghai Advanced Research Institute, Chinese Academy of Sciences, Shanghai 201203, China; bin.wu@sibcb.ac.cn (B.W.)
* Correspondence: ouyang@sibcb.ac.cn; Tel.: +86-021-54920143

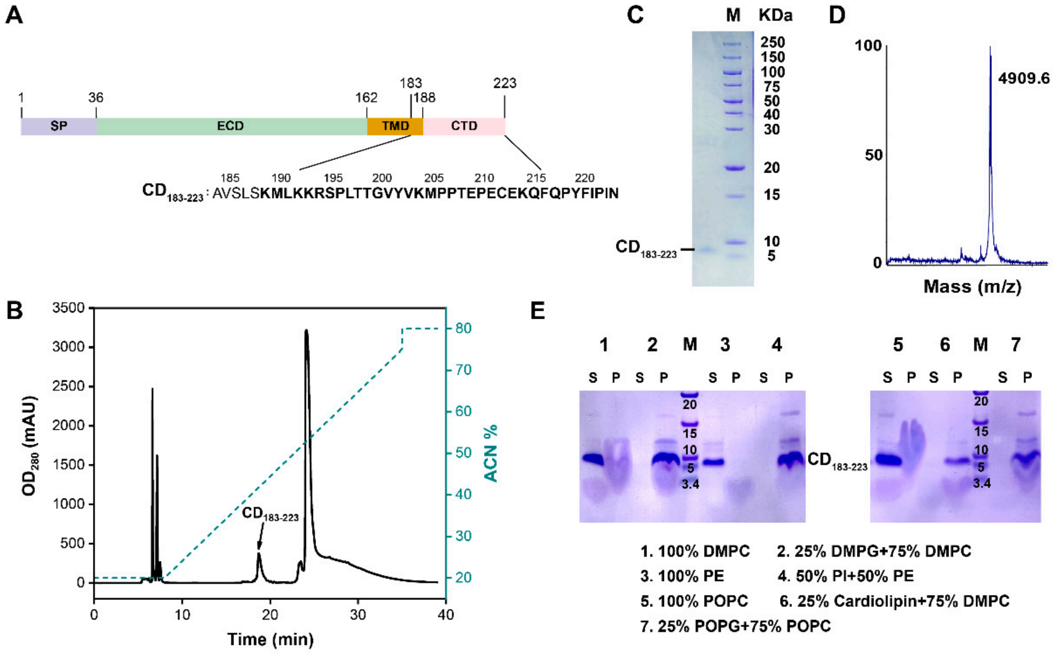

Figue S1. Expression and purification of $CD_{183–223}$. **(A)** $CD_{183–223}$ sequence (residues 183–223) used to prepare the fusion protein for NMR and biochemical experiments. **(B)** HPLC profile of $CD_{183–223}$ protein eluted with the gradient of Buffer B from 20% to 75% for 30 min and 80% for 5 min, respectively. The elution time, optical density at 280 nm (OD_{280}), and % of acetonitrile (ACN) in the elution buffer is indicated on the bottom, left and right axes, respectively. Black arrow indicates the $CD_{183–223}$ fraction. Buffer A: 100% (v/v) H_2O with 0.1% (v/v) trifluoroacetic acid (TFA); Buffer B: 100% (v/v) ACN and 0.1% (v/v) TFA. **(C)**

SDS-PAGE analysis of HPLC purified $CD_{183–223}$ protein. The lyophilized $CD_{183–223}$ powder was dissolved in gel loading buffer, and incubated at 100 °C for 5 min before SDS-PAGE. The $CD_{183–223}$ band was detected by Coomassie blue staining and indicated by an arrow. **(D)** Mass spectrometry analysis of HPLC purified $CD_{183–223}$ protein. The molecular weight determined for $CD_{183–223}$ is 4909.6 Da, consistent with the theoretical molecular weight of 4905.8 Da. **(E)** $CD_{183–223}$ was incubated with DMPC, POPC and PE liposomes supplemented with or without DMPG, cardiolipin, POPG and PI, respectively, as indicated on the bottom. The fractions from the pellet (P) and the supernatant (S) were separated by ultracentrifugation and analyzed by SDS-PAGE.

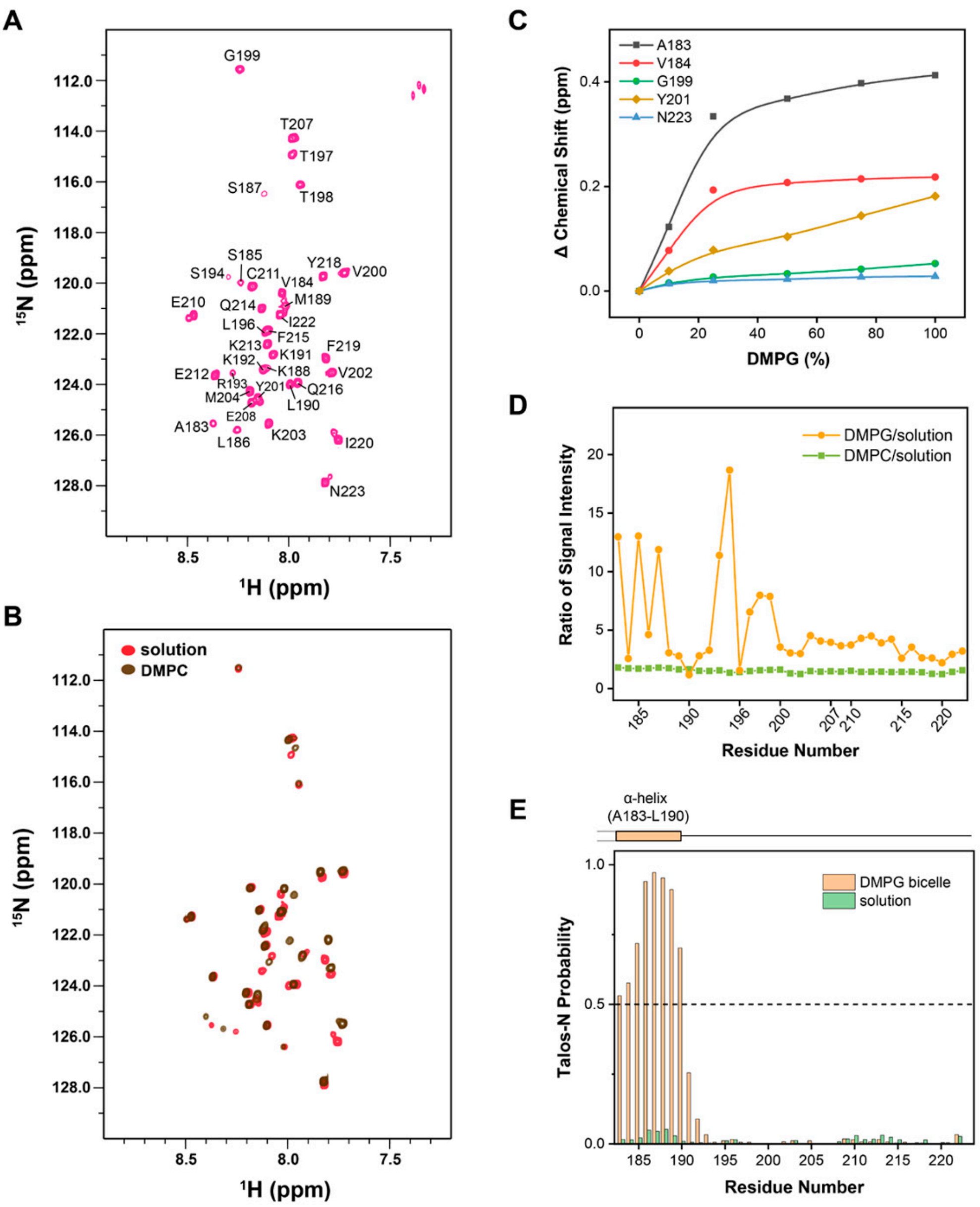

Figure S2. The NMR characterization in DMPC/DH^{6}PC bicelles and solution, and the lipid composition effects on $CD_{183–223}$. **(A)** ^1H-^{15}N TROSY-HSQC spectrum of $CD_{183–223}$ in buffer solution with backbone resonances assigned. The spectrum was recorded at ^1H frequency of 600 MHz using [^{15}N, ^{13}C]-labeled protein. **(B)** The comparison of 2D ^1H-^{15}N TROSY-

HSQC spectra of $CD_{183–223}$ in buffer solution (red) and in DMPC/DH^6PC (q = 0.7) bicelles (brown). **(C)** Plots of chemical shift changes versus DMPG percentage for partial residues. **(D)** Plots of intensity changes versus DMPG percentage for the cytoplasmic domain residues. **(E)** Chemical-shift-based secondary structure prediction of $CD_{183–223}$ in DMPG/DH^6PC bicelles (yellow) and in buffer solution (green). The graph shows the probability of each residue being part of the α-helix, as determined with TALOS+ software.

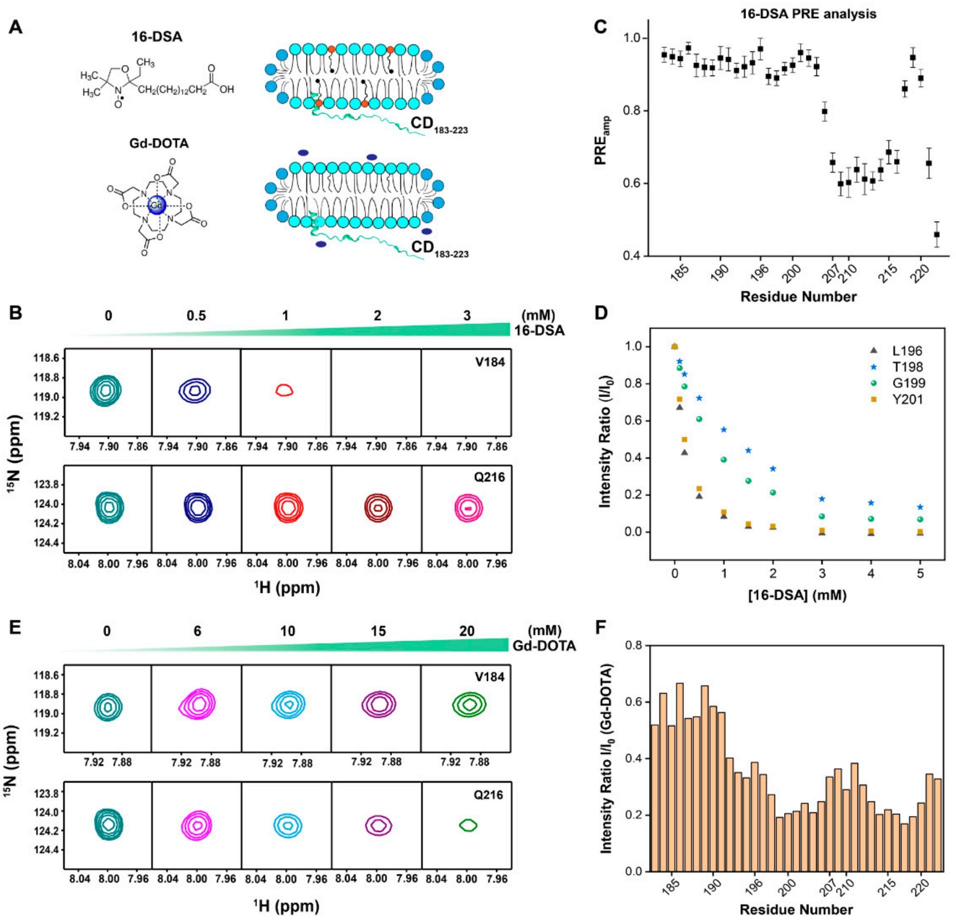

Figure S3. PRE analysis of CTLA-4 $CD_{183–223}$ partition into lipid bicelles. **(A)** Schematic illustration of titrating the bicelle-bounded $CD_{183–223}$ with the lipophilic PRE probe 16-DSA (orange balls) and water-soluble PRE probe Gd-DOTA (indigo balls). The black dots represent the nitroxide group in 16-DSA. **(B)** Parts of 2D ^1H-^{15}N TROSY-HSQC spectra of CTD in DMPG/DH^6PC bicelles measured at the indicated 16-DSA concentrations at ^1H frequency of 700 MHz NMR spectrometer at 30 °C. **(C)** PRE_{amp} versus residue number plot obtained from the 16-DSA titration. The peak intensity vs [16-DSA] data were fitted by the exponential decay function (Equation 1) to determine the PRE_{amp}. The results are represented as the PRE_{amp} values ± standard. **(D)** Residue-specific decay curves of four different residues, including L196, T198, G199 and Y201. **(E)** Parts of 2D ^1H-^{15}N TROSY-HSQC spectra of CTD in DMPG/DH^6PC bicelles measured at the indicated Gd-DOTA concentrations at ^1H frequency of 700 MHz NMR spectrometer at 30 °C. **(F)** Assessment of $CD_{183–223}$ in DMPG/DH^6PC bicelles from the PRE effects of hydrophilic Gd-DOTA paramagnetic probes. Peak intensity reductions induced by Gd-DOTA (20 mM) relative to a reference spectrum are shown as orange bar graphs.

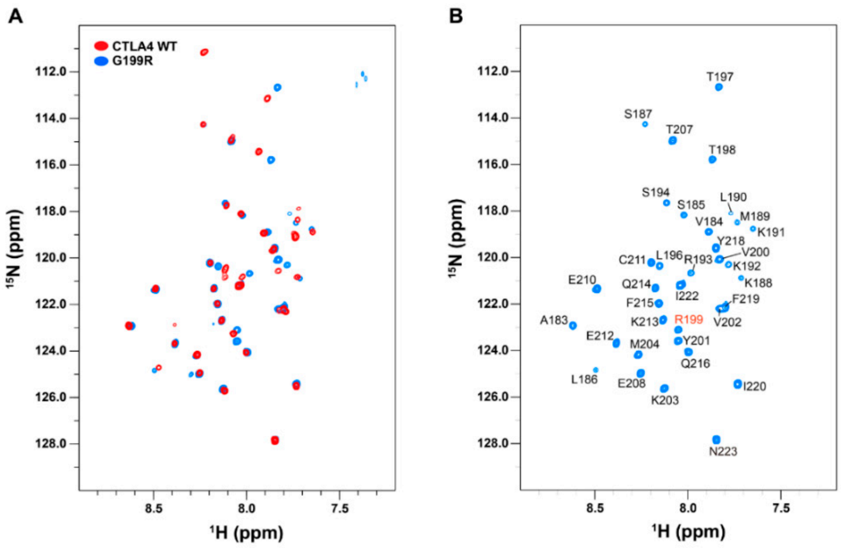

Figure S4. The NMR spectra of G199R mutant in bicelles. **(A)** The comparison of 2D ^{1}H-^{15}N TROSY-HSQC spectra of CD$_{183–223}$ (red) and G199R (blue) mutant in DMPG /DH^{6}PC (q=0.7) bicelles. **(B)** ^{1}H-^{15}N TROSY-HSQC spectrum of G199R mutant in DMPG/DH^{6}PC bicelles with backbone resonances assigned. The spectrum was recorded at 1H frequency of 600 MHz using [^{15}N, ^{13}C]-labeled protein.

Table S1. List of oligomers.

Name	Sequence
WT-F (CD)[1]	AGTGCTGTTTCAGGGCCCGGCTGTAAGTCTATCAAAAATG
WT-R (CD)	GGCTTTGTTAGCAGCCGGATCTTAGTTAATCGGGATGAAAT
Vector-F	ATTTCATCCCGATTAACTAAGATCCGGCTGCTAACAAAGCC
Vector-R	CATTTTTGATAGACTTACAGCCGGGCCCTGAAACAGCACT
G199R-F (CD)	CCGCTGACCACCCGTGTTTACGTGAAAAT
G199R-R (CD)	CATTTTCACGTAAACACGGGTGGTCAGCG
G199E-F	CCGCTGACCACCGAAGTTTACGTGAAAAT
G199E-R	TTTCACGTAAACTTCGGTGGTCAGCGGGC
WT-NotⅠ-F	AAGCGGCCGCATGGCTTGCCTTGGATTTCAG
WT-NheⅠ-R	TTGCTAGCTCAATTGATGGGAATAAAATAAGGCT
ΔC-NheⅠ-R	TTGCTAGCTCACATTTTGCTCAAAGAAACAG
G199R-F (FL)[2]	CCTCTTACAACACGGGTCTATGTGAAAATGC
G199R-R (FL)	CATTTTCACATAGACCCGTGTTGTAAGAGGG

[1] Constructs of CD$_{183–223}$ in *E.coli*. [2] Constructs of CTLA-4 full-length.
